# Supplementary material for: Opposing effects of HNP1 (α-defensin-1) on plasma cholesterol and atherogenesis
Source: PLoS One. 2020 Apr 17;15(4):e0231582. doi: 10.1371/journal.pone.0231582 (PMC7164655; doi:10.1371/journal.pone.0231582)
Supplement: S1 Data — (DOCX) [file pone.0231582.s001.docx]

**Supporting Information files**

**Materials.** Regular rodent chow diet (RD; 6.5% fat) and HFD (15.8% fat and 1.25% cholesterol) (TD.88051, Harlan) were purchased from Harlan (Harlan, Rehovot Israel) and cholestyramine from Sigma-Aldrich. α-def-1 was purchased from Sigma-Aldrich and kindly provided by Dr. Wuyan Lu (Univ. MD School of Medicine).  Each formed complexes with LDL *in vitro* and accelerated their clearance *in vivo*^12^ and were used interchangeably.

**Moderate high fat diet (MFD).** MFD was composed of 35 parts high fat diet (See attached left table) and 65 parts regular diet (See attached right table), a conventional approach used by commercial companies to prepare specific diets. This composition was chosen based on empirical evidence that it generated plasma levels of total cholesterol and LDL cholesterol comparable to plasma levels in animals treated with low dose of cholestyramine and because it led to formation of lipid streaks in the aortas of comparable size. We used this as a second independent control to animals exposed to a low dose of HNPs.


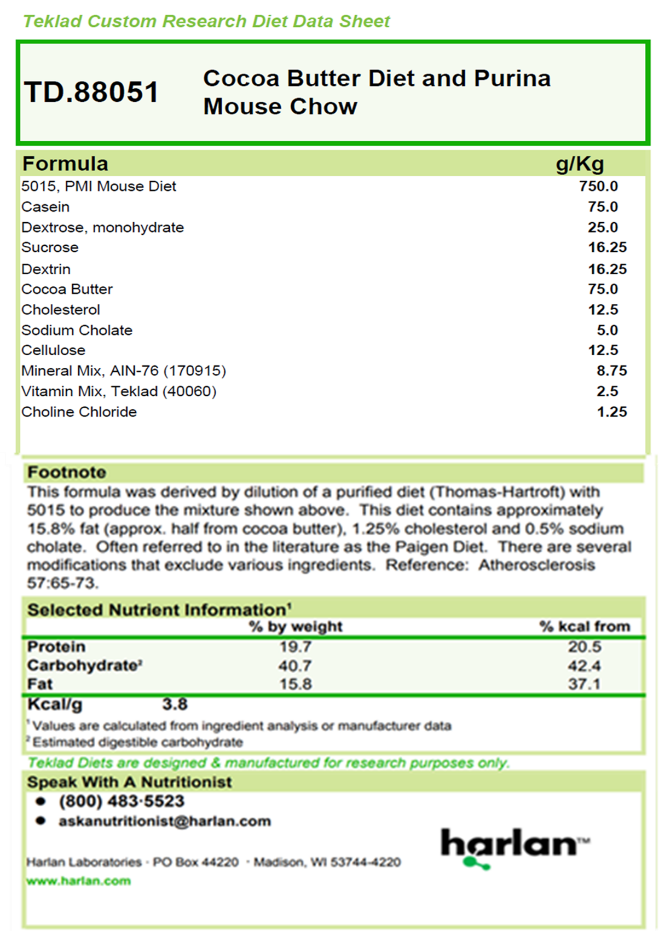


**
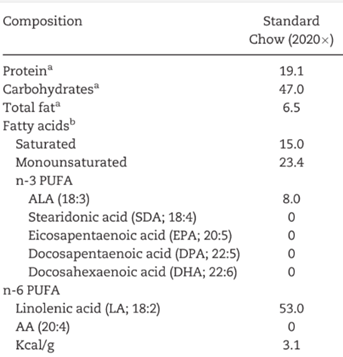
**

**Mice.** ApoE^-/-^ mice on a C57BL/6 background were bred in-house from a stock originating from Jackson Laboratories provided by M. Aviram (Rappaport Faculty of Medicine, Technion, Haifa, Israel). Animal care and experiments were conducted in accordance with protocols approved by the Animal Care Committee of the Hebrew University (approval number: MD-15-14579-4) and the University of Pennsylvania. Mice were maintained on a regular rodent chow diet, on an HFD, or a moderate high fat diet (MFD) by combining 65% RD with 35% HFD for the indicated times.

**Cholesterol-lowering.** Five approaches were used to modify plasma cholesterol in ApoE^−/−^ female mice (16 per group). One set of mice was fed a HFD for 6 weeks without or with 1.5% or 3% cholestyramine^18^. A second set of mice on a HFD, received an intravenous (IV) injection, via tail vein, of α-def-1 (10 or 30 μg) or vehicle (PBS) control every other day^13,19^. A sixth group, studied in parallel, was fed a MFD for 6 weeks. Mice were monitored for adverse effects throughout the experiment. There were no statistically significant differences in body weight between mice in any of the six groups. Mice were anesthetized with an intraperitoneal injection of zolazepam (25 mg/kg) and xylazine (50 mg/kg) on the last day of the experiment and blood samples were taken by transcardiac puncture after 6 hours of fasting^12^. Serum total cholesterol (TCH) and high-density lipoprotein cholesterol (HDL) were measured by enzymatic methods using an autoanalyser (Cobas 6000; Roche, Nakakojo, Japan), and levels of low-density lipoprotein cholesterol (LDL) were calculated as reported^12^.

**Staining of aortic roots.** After blood was withdrawn, mice were euthanized with pentobarbital and the hearts were immediately removed and transected midway between the apex and base in a plane parallel to a line defined by the tips of the atrial appendages. The basal ventricular segments in continuity with the atria and aortic roots were embedded in optimum cutting temperature compound (OCT) and frozen in liquid nitrogen. Cryostat sections were prepared at ~7 µm intervals using a CM 1900 cryotome (Leica Microsystems, Wetzlar, Germany), fixed in formalin, stained with Oil Red, and examined to assess proximity to the aortic root. Sections through the coronary ostia, coronary sinuses, and the aortic leaflets were captured. Lipid deposition was quantified in parallel sections stained with Oil Red, and quantified using Image-Pro Plus analysis software as previously reported^12^. Results are reported as the percentage of the circumference of each root that was Oil Red positive.
