# Supplementary material for: Opposing effects of HNP1 (α-defensin-1) on plasma cholesterol and atherogenesis
Source: PLoS One. 2020 Apr 17;15(4):e0231582. doi: 10.1371/journal.pone.0231582 (PMC7164655; doi:10.1371/journal.pone.0231582)
Supplement: Attachment — Submitted filename: Ansawring the revwieres 24-3-2020.docx [file pone.0231582.s003.docx]

Dr. Michael Bader
Academic Editor
PLOS ONE

Dear Dr. Bader:

We are writing in reference to MS: PONE-D-19-31479 “Opposing effects of HNP1 (α-defensin-1) on plasma cholesterol and atherogenesis” that we revised and are re-submitting to your journal.

We appreciate the efforts taken by the reviewers and the editor to examine our study in detail, consider it of merit for publication in PLOS ONE, and inviting us to submit a revised version of the manuscript.

In the revised version of our manuscript we addressed all the concerns raised by reviewer 2. We have corrected the errors noted by the reviewer and have made a sincere effort by a thorough re-reading to avoid any errors, including copyediting, in the revised text.

We hope that the revised manuscript is now suitable for publication in PLOS ONE.

Sincerely,

Abd Al-Roof Higazi, MD

For the authors
